# Abnormal resting-state functional connectivity of hippocampal subregions in type 2 diabetes mellitus-associated cognitive decline

**DOI:** 10.3389/fpsyt.2024.1360623

**Published:** 2024-09-23

**Authors:** Lin Yao, Meng-Yuan Li, Kang-Cheng Wang, Yan-Ze Liu, Hai-Zhu Zheng, Zhen Zhong, Shi-Qi Ma, Hong-Mei Yang, Meng-Meng Sun, Min He, Hai-Peng Huang, Hong-Feng Wang

**Affiliations:** ^1^ Institute of Acupuncture and Massage, Northeast Asian Institute of Traditional Chinese Medicine, Changchun University of Chinese Medicine, Changchun, Jilin, China; ^2^ College of Psychology, Shandong Normal University, Jinan, Shandong, China; ^3^ Acupuncture and Tuina Center, The Third Affiliated Clinical Hospital of Changchun University of Chinese Medicine, Changchun, Jilin, China; ^4^ College of Acupuncture and Massage, Changchun University of Chinese Medicine, Changchun, Jilin, China

**Keywords:** type 2 diabetes mellitus, cognitive decline, resting-state functional connectivity, hippocampus, subregion; peripheral inflammation

## Abstract

**Objective:**

Type 2 diabetes mellitus (T2DM) over time predisposes to inflammatory responses and abnormalities in functional brain networks that damage learning, memory, or executive function. The hippocampus is a key region often reporting connectivity abnormalities in memory disorders. Here, we investigated peripheral inflammatory responses and resting-state functional connectivity (RSFC) changes characterized of hippocampal subregions in type 2 diabetes-associated cognitive decline (T2DACD).

**Methods:**

The study included 16 patients with T2DM, 16 patients with T2DACD and 25 healthy controls (HCs). Subjects were assessed for cognitive performance, tested for the expression of inflammatory factors IL-6, IL-10 and TNF-α in peripheral serum, underwent resting-state functional magnetic resonance imaging scans, and analyzed for RSFC using the hippocampal subregions as seeds. We also calculated the correlation between cognitive performance and RSFC of hippocampal subregion, and analyzed the significantly altered RSFC values of T2DACD for Receiver Operating Characteristic (ROC) analysis.

**Results:**

T2DACD patients showed a decline in their ability to complete cognitive assessment scales and experimental paradigms, and T2DM did not show abnormal cognitive performance. IL-6 expression was increased in peripheral serum in both T2DACD and T2DM. Compared with HCs, T2DACD showed abnormalities RSFC of the left anterior hippocampus with left precentral gyrus and left angular gyrus. T2DM showed abnormalities RSFC of the left middle hippocampus with right medial frontal gyrus, right anterior and middle hippocampus with left precuneus, left anterior hippocampus with right precuneus and right posterior middle temporal gyrus. Compared with T2DM, T2DACD showed abnormalities RSFC of the left posterior hippocampus and right middle hippocampus with left precuneus. In addition, RSFC in the left posterior hippocampus with left precuneus of T2DACD was positively correlated with Flanker conflict response time (r=0.766, *P*=0.001). In the ROC analysis, the significantly altered RSFC values of T2DACD achieved significant performance.

**Conclusions:**

T2DACD showed a significant decrease in attentional inhibition and working memory, peripheral pro-inflammatory response increased, and abnormalities RSFC of the hippocampal subregions with default mode network and sensory-motor network. T2DM did not show a significant cognitive decline, but peripheral pro-inflammatory response increased and abnormalities RSFC of the hippocampus subregions occurred in the brain. In addition, the left precuneus may be a key brain region in the conversion of T2DM to T2DACD. The results of this study may provide a basis for the preliminary diagnosis of T2DACD.

## 1 Introduction

Due to insufficient insulin secretion, type 2 diabetes mellitus (T2DM) patients are in a state of hyperglycemia for a long time, which predisposes them to chronic damage and dysfunction of blood vessels, nerves and brain (1–3). Cognitive dysfunction is a common complication of T2DM (4). Multiple meta-analyses of the association between T2DM and dementia across all ages have reported risk ratios between 1.43 and 1.62 (5–8). A longitudinal cohort study showed that the younger age at onset of diabetes, the greater risk of dementia (9). Type 2 diabetes mellitus-associated cognitive decline (T2DACD) mainly affects learning and memory, mental flexibility and mental speed (10). Without timely and effective intervention, cognitive abilities may gradually deteriorate and progress to dementia (11). Therefore, early identification and detection of changes in T2DACD may help clinicians to prevent severe cognitive decline.

Pathological changes in T2DACD include brain atrophy, neurofibrillary tangles, senile plaques, neuroinflammation, and local neuronal apoptosis (12, 13). Although T2DACD is a complex central nervous system disease caused by multi-component, multi-target and multi-channel effects, the functional connectivity (FC) network abnormality of central system may be an important pathological basis (14). In the central nervous system, the hippocampus is the neurobiological basis of cognition, memory, inhibition, emotion and other functions, and participates in a variety of neural circuits (such as limbic system, neocortex, etc.) (15). Clinical and animal studies have shown that the hippocampus can be divided into three parts along its long axis: head, body and tail (i.e. anterior, middle and posterior). There are some differences in gene expression, anatomical structure and function of each part (16–19). The research found that the posterior hippocampus is primary FC with sensory-motor, middle with default mode network (DMN), and anterior with limbic networks and prefrontal (20). The anterior hippocampus is involved in emotion and affect, and the middle/posterior involved in cognitive functions such as spatial and episodic memory (17, 21, 22). During the development of Alzheimer’s disease (AD), the hippocampus is one of the first region of brain atrophy (23). In the brains of patients with cognitive impairment, there are also extensive FC abnormalities in the hippocampus. In patients with AD, there are FC abnormalities between the hippocampus and several brain regions, including medial prefrontal cortex, ventral anterior cingulate cortex, posterior cingulate cortex, right inferior temporal gyrus and right superior middle temporal gyrus (24). In addition, research has found that there are abundant insulin receptors in the hippocampus (25), which is more susceptible to central insulin resistance than other brain regions (26, 27). Hippocampal insulin resistance is an important mechanistic mediator of cognitive impairment in T2DM and AD (26). Previous MRI studies on diabetes patients have provided evidence that cognitive impairment is related to changes in hippocampal structure and function (28, 29). Therefore, some people believe that hippocampal abnormalities in T2DM may disrupt the upstream and downstream pathways responsible for memory signal transmission and regulation, and explain the reasons for cognitive impairment in T2DM (29). However, existing research mostly focuses on FC analysis of the hippocampus as a whole, and there is relatively little exploration of the anterior, middle, and posterior zoning of the hippocampus as an interesting study of FC changes.

It is worth noting that peripheral inflammation has been described as a potential risk factor for AD and vascular dementia (30, 31). Several meta-analyses have found that compared to individuals with normal neurological function, inflammatory proteins such as interleukin-1β(IL-1β), IL-6, IL-10, TNF-α, high-sensitivity C-reactive protein are elevated in the blood of AD patients (30, 32). Cognitive normal individuals with elevated inflammatory markers in blood have a higher risk of developing mild cognitive impairment (MCI), AD dementia, and all-cause dementia in the future (33). Studies have shown that peripheral inflammatory responses can promote a pro-inflammatory environment in the central nervous system by signaling through the blood-brain barrier, endothelial cells, or periventricular organs, and stimulating the vagus nerve (which signals the detection of inflammatory proteins through direct afferent connections to the brainstem), thereby triggering or exacerbating neurodegenerative processes that ultimately lead to cognitive decline and dementia (34). Although inflammatory responses have been associated with the development of cognitive dysfunction, little is known about whether peripheral inflammatory signaling affects the connectivity of central networks. A review of studies of this relationship in several clinical populations found that pro-inflammatory cytokines, specifically IL-6 and CRP, were associated with decreased white matter integrity and gray matter volume in healthy older adults (35). Elevated blood inflammatory markers in middle-aged adults are associated with accelerated cognitive aging (36). These findings may speculate a possibility that peripheral inflammatory signaling may cause abnormal neural activation and abnormal functional connections between brain regions, leading to cognitive impairment. However, there is currently limited research on the changes in peripheral inflammation of T2ACD, and it is still in the preliminary exploration stage.

Considering the structural complexity of the hippocampus and its potential role in diseases related to cognitive decline, as well as the impairment of cognitive ability caused by peripheral inflammation. In this study, we used a seed-based resting-state functional connectivity (RSFC) approach to explore the RSFC patterns of the hippocampus and use high-sensitivity ELISA kits to measure the peripheral inflammation of T2DACD for preliminary detection. Our primary hypothesis is that in T2DCD, the hippocampal subregion and other cortical regions exhibit abnormal RSFC patterns, accompanied by heightened expression of peripheral inflammatory factors. Furthermore, we aim to explore the association between RSFC changes in hippocampal subregions and the clinical characteristics of T2DCAD, and assess the predictive value of RSFC patterns in the hippocampal subregions to other cortical regions, thereby garnering crucial information with potential clinical implications.

## 2 Materials and methods

### 2.1 Participants

All subjects were recruited from the outpatient department, inpatient department and community of acupuncture and Massage Center of the Third affiliated hospital of Changchun University of Chinese Medicine, and had signed an informed consent form. The subjects include 16 patients with T2DM, 16 patients with T2DACD and 25 healthy controls (HCs). All subjects are right-handed, aged between 50 and 70 years, and possessed > 6 years of education. T2DM diagnosis is based on the 2022 American Diabetes Association Standards for the Medical Care of Diabetes (37), and the course of disease should be ≥ 2 years. Patients with cognitive impairment were scored of <26 on the Montreal Cognitive Assessment Scale (MoCA) (38, 39). Subjects in the T2DM group reported using oral hypoglycemic medications and/or insulin for glycemic control (eight with one oral hypoglycemic medication or insulin, one with two or more hypoglycemic medications, three with both medications and insulin, and four without hypoglycemic medication or insulin). Subjects in the T2DACD group reported using oral hypoglycemic medications and/or insulin for glycemic control (three with one oral hypoglycemic medication or insulin, five with two or more hypoglycemic medications, seven with both medications and insulin, and four without hypoglycemic medication or insulin).

This study was approved by the Medical Ethics Committee of the third Affiliated Hospital of Changchun University of Chinese Medicine (Ethics Committee Approval Document No.: CZDSfYLL2020-001-01). All participants had signed an informed consent form before enrollment.

### 2.2 Glycolipid metabolism indicators and inflammation factors measures

Blood samples from T2DM, T2DACD, and HCs were obtained in the morning under fasting conditions without alcohol consumption for at least one day prior to testing. Through venipuncture, fasting blood was drawn from each subject for the detection of glycolipid metabolism indicators and inflammation factors. Glycolipid metabolism indicators included blood glucose (fasting blood glucose and glycated hemoglobin), and blood lipid(triglycerides, total cholesterol, high-density lipoprotein cholesterol and low-density lipoprotein cholesterol) were applied with a fully automated biochemical analyzer. Concentrations of the inflammatory factors IL-6 (Huangshi R&C Biotechnology Co., Ltd., Product No. SU-B10377), IL-10 (Huangshi R&C Biotechnology Co., Ltd., Product No. SU-B13626), and TNF-alpha (Huangshi R&C Biotechnology Co., Ltd., Product No. SU-B11776) in the peripheral serum were measured using high-sensitivity ELISA kits, which were used according to the manufacturer’s instructions.

### 2.3 Cognitive and emotional assessment

Cognitive function assessment included MoCA, Lateral Inhibition Task (Flanker paradigm), Cognitive Conflict Task (Stroop paradigm) and Working Memory Task (N-back paradigm). Emotional assessment included Self-Rating Anxiety Scale (SAS), Self-rating depression scale (SDS) and Hamilton Depression Scale (HAMD).

### 2.4 Image acquisition

A Siemens 3.0T high-field intensity MRI scanner (model: 3TMAGNETO Trio) was used to perform MRI scans on the subjects. All participants were instructed to remain awake and eyes closed. ①3D-T1 high-resolution structural images were acquired using MPRAGE sequence[Slices: 176; TR: 2300ms; TE: 3.33ms; Slice Thickness: 1.0mm; FOV: 256mm×256mm; Voxel size: 1.0mm×1.0mm×1.0mm; Data acquisition time: 9:50s]. ②BOLD image uses an echo EPI sequence[Slices:32;TR:3000ms;TE:30ms;Slice Thickness:3.0mm;FOV:220mm×220mm;Voxel size:3.4mm×3.4mm×3.0mm;Data acquisition time:6:23s]. Each subject scanned at least 3 runs to ensure the stability of BOLD data.

### 2.5 Data Preprocessing

**Figure 1:**
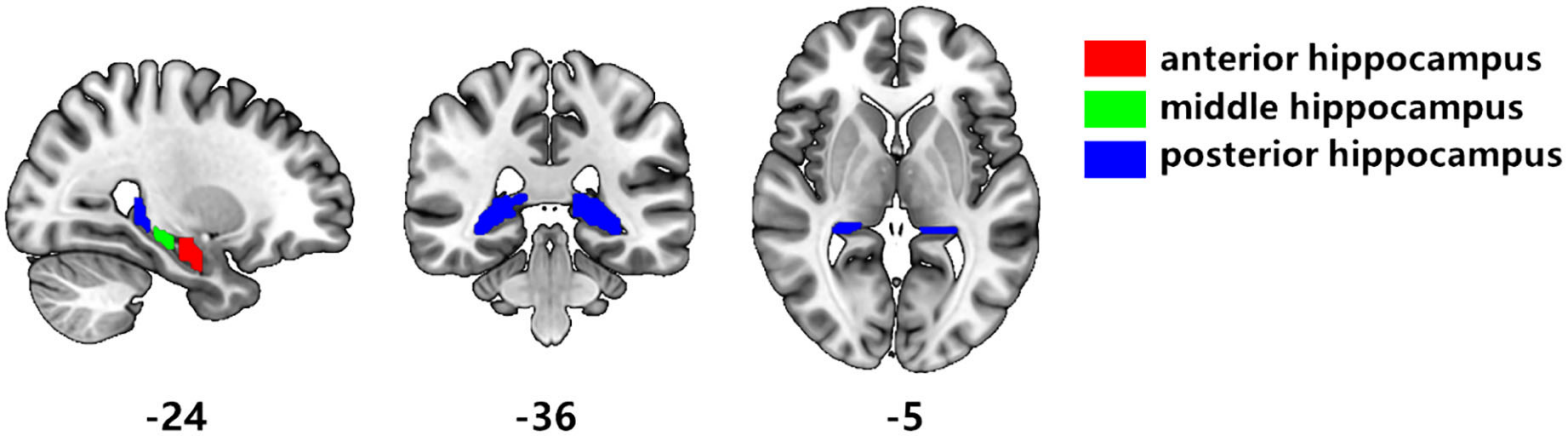
Segmentation of hippocampal subregions. According to the results of Hu et al. [40], the hippocampus is divided segmented three subregions. Red is the anterior hippocampus, green is the middle hippocampus, and blue is the posterior hippocampus.

First, the DICOM format data is converted to NifTI (nii) format by using MRIcroGL software. Then use FSL and SPM 12 for preprocessing. Delete the first four time points of each run for functional image data, time layer correction, head movement correction, spatial standardization (using the MNI standard template of FSL), smoothing filtering (using 6mm half-height full-width Gaussian smoothing kernel to conduct convolution operation with image data), linear drift removal, bandwidth filtering (0.01-0.08Hz). The anterior, middle and posterior hippocampal subregions were taken as regions of interest for whole brain functional connectivity analysis (40) (**Figure 1**). Calculate the average time series of each seed, and the correlation coefficient between each seed and the time series of other voxels in the whole brain. Then, perform Fisher’s r-z transformation to obtain the functional connectivity matrix of brain regions.

### 2.6 Statistical Analysis

#### 2.6.1 Statistics of clinical indicator data

The statistical analysis of demographic data, glycolipid metabolism indicators, cognitive and emotional ability were conducted using IBM SPSS Statistics 26 software (IBM, Armonk, NY, United States). For assessment whether T2DACD patients had cognitive decline, the One-way ANOVA, *post hoc* test and online software package MetaboAnalyst 5.0 (https://www.metaboanalyst.ca/) were used to assess the differences among the three groups during the baseline period.

#### 2.6.2 Functional connectivity analysis

To observe the RSFC changes in hippocampal subregions of T2DACD at baseline, SPM12 was used to conduct One-way ANOVA and *post hoc* test for the three groups (HCs, T2DACD and T2DM). The union template was used as a mask, and age and gender were used as covariate regression. The initial statistical threshold was the voxel level *p*<0.001 (uncorrected), and the multiple comparison correction adopted the cluster level FWE, *p*<0.05 was considered statistically significant, so as to obtain the F value plots with significant differences between the three groups and the T value plots with significant differences between the two groups.

#### 2.6.3 Correlation analysis

We explored the relationships of T2DACD between RSFC value of different brain regions and the scores of cognitive function assessment. Pearson partial correlation analysis was conducted with age, gender, and education level as control variables (*p*<0.05) to determine the directly related brain areas of cognitive function decline.

#### 2.6.4 Receiver operating characteristic analysis

The significantly altered RSFC z-values of hippocampal subregion seeds compared T2DACD with HCs and T2DACD with T2DM extracted and used for the Receiver Operating Characteristic (ROC) analysis using GraphPad PRISM version 9.5(GraphPad Software—San Diego, CA, USA). Sensitivity, specificity, area under the curve (AUC), 95% confidence interval (CI), and maximum Youden’s index (sensitivity+specificity -1) (41) were calculated for the ROC curves for each changed brain region.

## 3 Results

### 3.1 Demographics, clinical, cognitive and emotional characteristics

**Figure 2:**
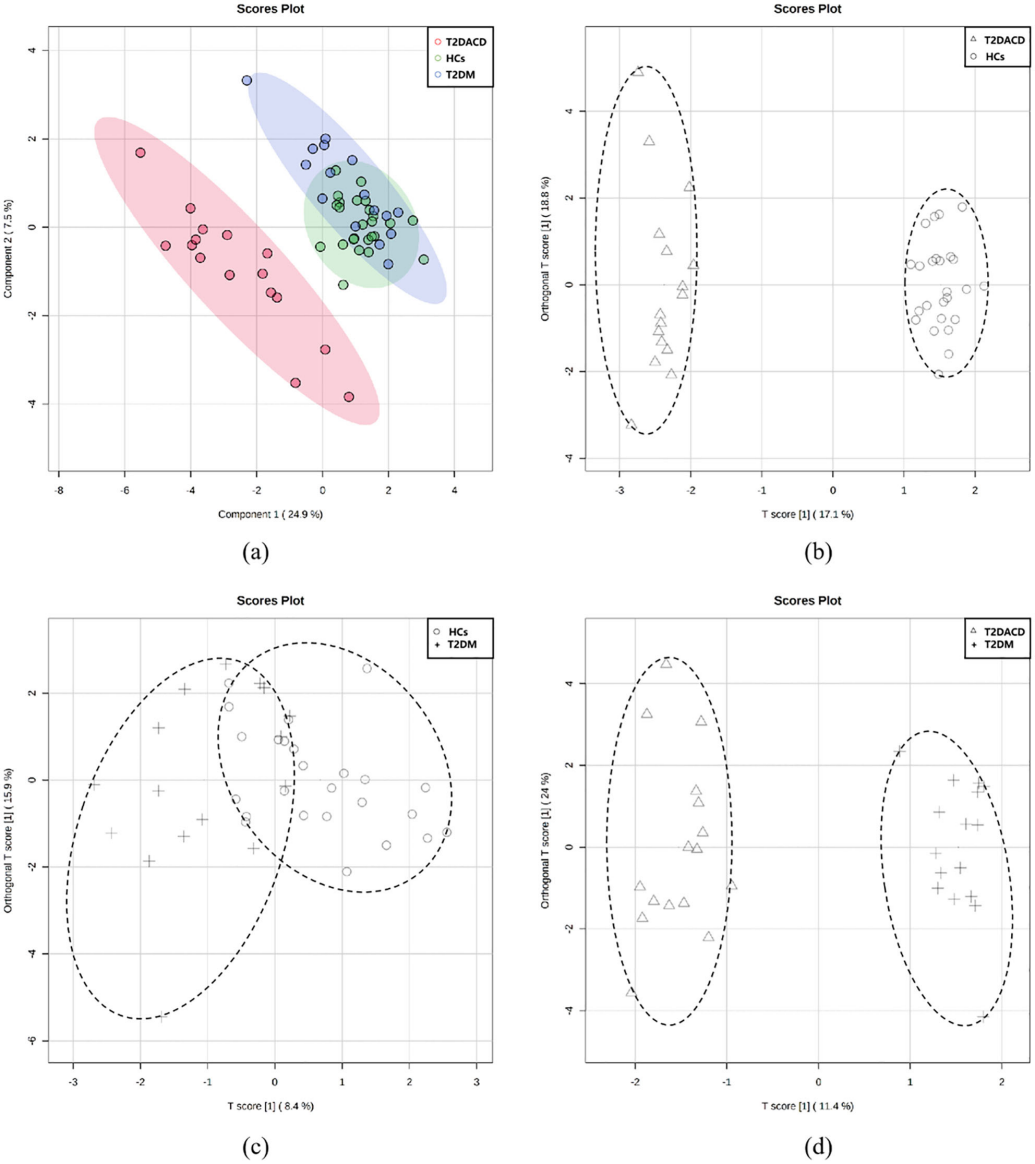
**(A) **PLS-DA models of three groups; **(B–D)** pairwise grouping of the first two major components of the score chart was analyzed based on OPLS-DA; **(B)** T2DACD vs. HCs: R2Y = 0.985, Q2 = 0.937, iterative permutation test *p* < 0.01; **(C)** T2D vs. HCs: R2Y = 0.442, Q2 = -0.177, iterative permutation test *p* = 0.27; **(D)** T2DACD vs. T2D: R2Y = 0.968, Q2 = 0.901, iterative permutation test *p* < 0.01. HCs, health control; T2DM, type 2 diabetes mellitus; T2DACD, type 2 diabetes mellitus-associated cognitive decline.

**Table 1** summarizes the demographic and glycolipid metabolism indicators of three groups. There were no differences among the three groups in age, gender, and years of education. FPG and HbA1c in T2DACD and T2DM were significantly higher than HCs, and no differences in other lipid metabolism indicators. **Table 2** summarizes the results of the peripheral serum inflammation factors in the three groups. Peripheral IL-6 levels were significantly higher in T2DM and T2DACD than HCs, but there was no difference in IL-10 and TNF-α. **Table 3** summarizes the cognitive and emotional characteristics of three groups. T2DACD patients had significantly lower MoCA scores than HCs and T2DM. T2DACD also performed significantly worse than HCs and T2DM on the lateral inhibition task (Flanker conflict response), cognitive conflict task (Stroop neutrality response, Stroop conflict response), and working memory task (1-back response). There were no differences in emotional assessment indicators among three groups. In **Figure 2**, the PLS-DA model diagram was used to visualize the natural distribution of all cognitive assessment indicators among three groups in a three-dimensional space, indicating that the T2DACD could be well separated from T2DM and HCs, while the T2DM overlaps with HCs.

**Table 1:** Demographic and glycolipid metabolism indicators data for T2DM, T2DACD and HCs.

Record Variable	Group			HCs vs.T2DM vs. T2DACD	HCs vs.T2DM	HCs vs.T2DACD	T2DM vs.T2DACD
	HCs (n=25)	T2DM (n=16)	T2DACD(n=16)	*p* Value			
Age (years)	55.96 ± 5.48	59.50 ± 7.07	58.75 ± 4.81	0.126	—	—	—
Gender (male/female)*	7/18	5/11	9/7	—	—	—	—
Education (years)	11.56 ± 2.47	12.25 ± 3.00	11.81 ± 2.48	0.716	—	—	—
Duration of diabetes (years)	—	11.00 ± 7.94	10.81 ± 7.08	—	—	—	—
Height (m)	1.63 ± 0.06	1.65 ± 0.06	1.67 ± 0.07	0.209	—	—	—
Weight (kg)	63.34 ± 6.80	65.19 ± 7.55	68.00 ± 8.70	0.168	—	—	—
FPG	5.53 ± 0.24	7.70 ± 1.37	8.79 ± 3.63	**<0.001**	**0.002**	**<0.001**	0.137
HbA1c	5.58 ± 0.19	6.68 ± 0.58	7.68 ± 1.84	**<0.001**	**0.001**	**<0.001**	**0.008**
TC	4.87 ± 1.23	4.93 ± 0.97	5.21 ± 0.77	0.552	—	—	—
TG	1.83 ± 1.44	1.63 ± 0.61	2.12 ± 1.45	0.547	—	—	—
HDL-C	1.27 ± 0.22	1.22 ± 0.36	1.13 ± 0.17	0.252	—	—	—
LDL-C	3.10 ± 0.92	3.26 ± 1.05	3.58 ± 0.93	0.311	—	—	—

Data are mean ± SD unless otherwise stated. Statistically significant results (i.e., p<0.05) appear in boldface type. * P value for χ^2^ test. HCs, health controls; T2DM, type 2 diabetes mellitus; T2DACD, type 2 diabetes mellitus-associated cognitive decline; FPG, fasting plasma glucose/fasting blood glucose; HbA1c, Hemoglobin A1C; TC, total cholesterol; TG, triglyceride; HDL-C, high-density lipoprotein; LDL-C, low-density lipoprotein.

**Table 2:** Inflammation factors data for T2DM, T2DACD and HCs (pg/mL).

Record Variable	Group			HCs vs.T2DM vs. T2DACD	HCs vs.T2DM	HCs vs.T2DACD	T2DM vs.T2DACD
	HCs (n=10)	T2DM (n=8)	T2DACD(n=10)	*p* Value			
IL-6	0.160 ± 0.047	0.166 ± 0.003	0.168 ± 0.003	**<0.001**	**0.005**	**<0.001**	0.234
IL-10	0.087 ± 0.001	0.087 ± 0.002	0.087 ± 0.003	0.976	—	—	—
TNF-α	0.237 ± 0.004	0.237 ± 0.003	0.238 ± 0.009	0.866	—	—	—

Data are mean ± SD unless otherwise stated. Statistically significant results (i.e., p<0.05) appear in boldface type. * P value for χ^2^ test. HCs, health controls; T2DM, type 2 diabetes mellitus; T2DACD, type 2 diabetes mellitus-associated cognitive decline.

**Table 3:** cognitive and emotional assessment data.

Record Variable	Group			HCs vs.T2DM vs. T2DACD	HCs vs.T2DM	HCs vs.T2DACD	T2DM vs.T2DACD
	HCs (n=25)	T2DM (n=16)	T2DACD(n=16)	*P* Value			
MoCA	27.32 ± 1.18	26.81 ± 0.91	23.38 ± 1.71	**<0.001**	0.224	**<0.001**	**<0.001**
Flanker-no conflict							
reaction time (s)	0.62 ± 0.08	0.64 ± 0.10	0.70 ± 0.15	0.090	—	—	—
accuracy rate	0.97 ± 0.05	0.95 ± 0.04	0.94 ± 0.03	0.174	—	—	—
Flanker-conflict							
reaction time (s)	0.67 ± 0.08	0.69 ± 0.09	0.76 ± 0.16	**0.048**	0.475	**0.015**	0.110
accuracy rate	0.93 ± 0.06	0.92 ± 0.11	0.89 ± 0.06	0.274	—	—	—
Stroop-neutrality							
reaction time (s)	0.83 ± 0.12	0.88 ± 0.16	1.02 ± 0.25	**0.006**	0.457	**0.002**	**0.025**
accuracy rate	0.97 ± 0.05	0.93 ± 0.12	0.91 ± 0.16	0.266	—	—	—
Stroop-consistency							
reaction time (s)	0.84 ± 0.13	0.88 ± 0.20	0.99 ± 0.21	0.051	—	—	—
accuracy rate	0.96 ± 0.08	0.92 ± 0.09	0.92 ± 0.11	0.243	0.167	0.156	0.971
Stroop-conflict							
reaction time (s)	0.93 ± 0.12	0.94 ± 0.23	1.10 ± 0.23	**0.025**	0.920	**0.011**	**0.026**
accuracy rate	0.92 ± 0.08	0.89 ± 0.13	0.83 ± 0.13	0.075	—	—	—
1-back							
reaction time (s)	0.75 ± 0.12	0.86 ± 0.18	0.94 ± 0.15	**<0.001**	0.052	**<0.001**	0.135
accuracy rate	0.90 ± 0.07	0.89 ± 0.09	0.84 ± 0.08	0.075	—	—	—
2-back							
reaction time (s)	1.11 ± 0.29	1.17 ± 0.33	1.32 ± 0.38	0.178	—	—	—
accuracy rate	0.74 ± 0.07	0.76 ± 0.09	0.74 ± 0.08	0.718	—	—	—
SAS	28.36 ± 0.61	29.13 ± 0.61	30.31 ± 1.11	0.200	—	—	—
SDS	27.12 ± 0.48	26.63 ± 0.40	28.25 ± 1.21	0.324	—	—	—
HAMD	4.04 ± 0.28	4.25 ± 0.36	4.06 ± 0.31	0.882	—	—	—

Data are mean ± SD unless otherwise stated. Statistically significant results (i.e., p<0.05) appear in boldface type. HCs, health controls; T2DM, type 2 diabetes mellitus; T2DACD, type 2 diabetes mellitus-associated cognitive decline; MoCA, Montreal Cognitive Assessment; SAS, Self-Rating Anxiety Scale; SDS, Self-rating depression scale; HAMD, Hamilton Depression Rating Scale.

### 3.2 Resting-state functional connectivity

**Figure 3:**
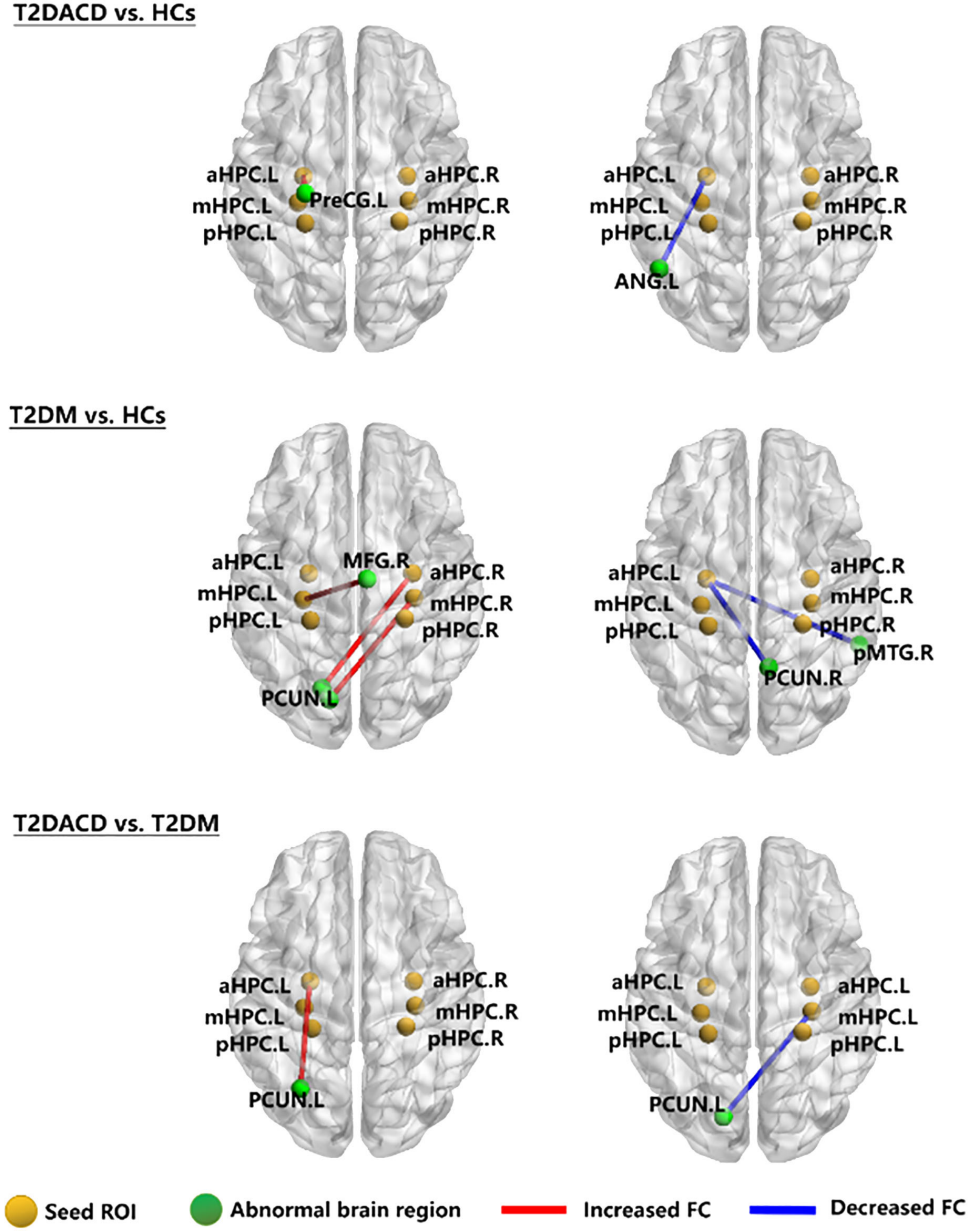
The RSFC network maps. HCs, healthy controls; T2DM, type 2 diabetes mellitus; T2DACD, type 2 diabetes mellitus-associated cognitive decline; aHPC.L, left anterior hippocampus; aHPC.R, right anterior hippocampus; mHPC.L, left middle hippocampus; mHPC.R, right middle hippocampus; pHPC.L, left posterior hippocampus; pHPC.R, right posterior hippocampus; PreCG.L, left precentral gyrus; ANG.L, left angular gyrus; PCUN.L, left precuneus; PCUN.R, rightprecuneus; MFG.R, right medial frontal gyrus; pMTG.R, right posterior middle temporal gyrus.

**Figure 4:**
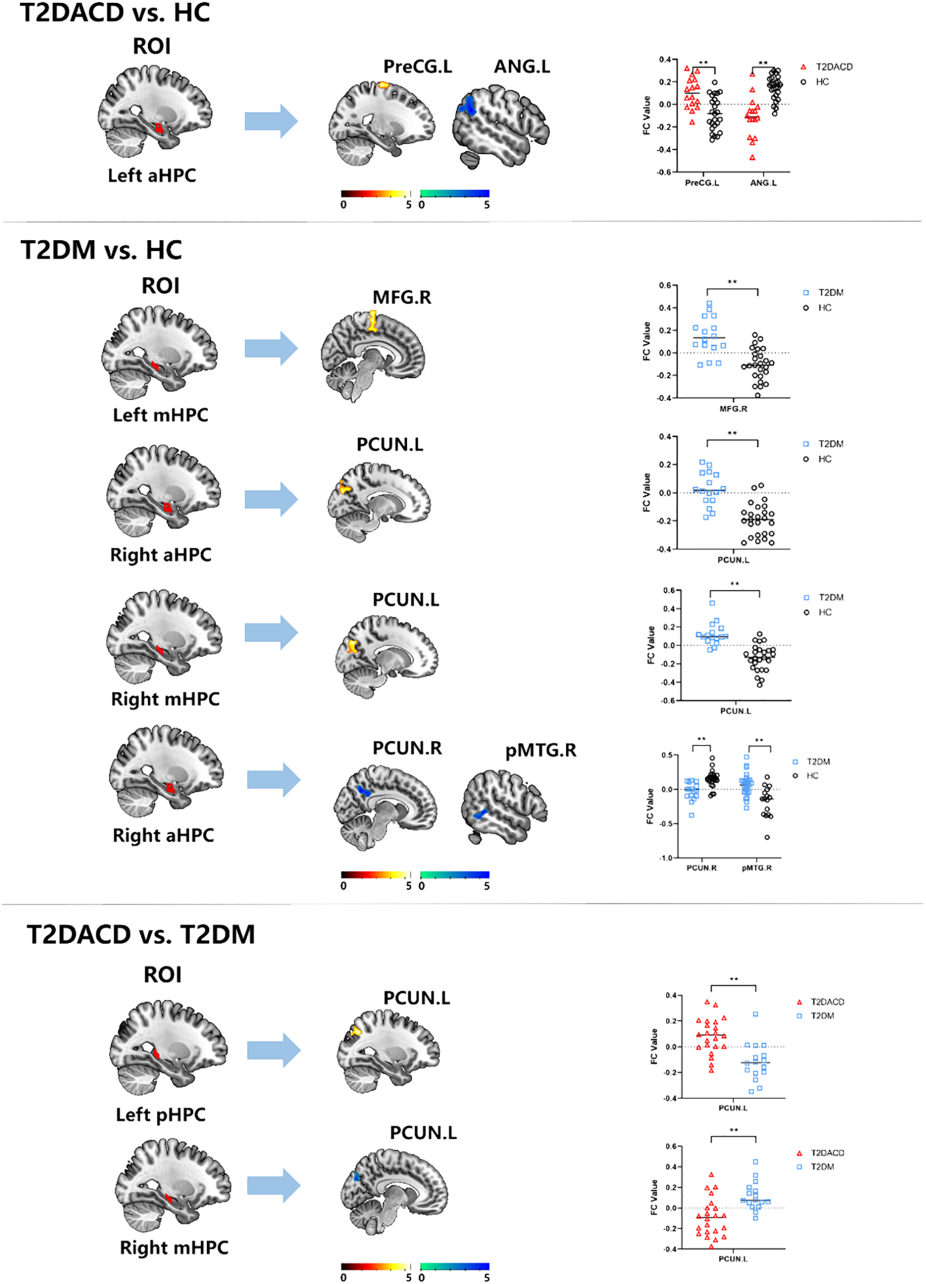
The RSFC results of hippocampal subregions and different brain regions between T2DACD and HC, T2DM and HC, T2DACD and T2DM. The bar chart shows the quantitative comparison of functional connectivity in these regions. HCs, healthy controls; T2DM, type 2 diabetes mellitus; T2DACD, type 2 diabetes mellitus-associated cognitive decline; aHPC.L, left anterior hippocampus; aHPC.R, right anterior hippocampus; mHPC.L, left middle hippocampus; mHPC.R, right middle hippocampus; pHPC.L, left posterior hippocampus; pHPC.R, right posterior hippocampus; PreCG.L, left precentral gyrus; ANG.L, left angular gyrus; PCUN.L, left precuneus; PCUN.R, rightprecuneus; MFG.R, right medial frontal gyrus; pMTG.R, right posterior middle temporal gyrus. **P < 0.01.

The results of the three groups analyses showed that the RSFC of the left anterior hippocampus with bilateral angular gyrus and the right anterior/middle hippocampus with left precuneus were changed. The *post hoc* test showed that compared with HCs, T2DACD showed increased RSFC of the left anterior hippocampus with left precentral gyrus and decreased RSFC of the left anterior hippocampus with left angular gyrus. Compared with HCs, T2DM showed increased RSFC of the left middle hippocampus with right medial frontal gyrus, right anterior hippocampus with left precuneus and right middle hippocampus with left precuneus. T2DM showed decreased RSFC of the left anterior hippocampus with right precuneus and right posterior middle temporal gyrus. Compared with T2DM, T2DACD showed increased RSFC of the left posterior hippocampus with left precuneus and decreased RSFC of the right middle hippocampus with left precuneus (**Figures 3**, **4**, **Tables 4**, **5**. *p*<0.05, corrected with FWE).

**Table 4:** Significant differences in seed-based resting-state functional connectivity among three groups.

Seed	Brain area	Voxel numbers	MNI(mm)			F value
			x	y	z	
Left aHPC	Left angular gyrus	169	-64	-54	30	14.15
	Right angular gyrus	99	32	-76	50	15.40
Right mHPC	Left precuneus	124	-14	-78	34	15.56
Right aHPC	Left precuneus	82	-18	-72	34	16.62

All tests were corrected for multiple comparisons using cluster-level FWE corrected at *p*<0.05, voxel-level uncorrected at *p*<0.001, voxels size>50. HCs, healthy controls; T2DM, type 2 diabetes mellitus; T2DACD, type 2 diabetes mellitus-associated cognitive decline; aHPC, anterior hippocampus; mHPC, middle hippocampus.

**Table 5:** Significant differences in seed-based resting-state functional connectivity between groups.

Comparison	Seed	Brain area	Voxel numbers	MNI(mm)			T value
				x	y	z	
T2DACD>HCs	Left aHPC	Left precentral gyrus	139	-24	-16	72	4.92
T2DACD<HCs	Left aHPC	Left angular gyrus	207	-50	-60	34	-4.67
T2DM>HCs	Left mHPC	Right medial frontal gyrus	167	6	-14	66	4.55
	Right aHPC	Left precuneus	202	-14	-78	34	4.86
	Right mHPC	Left precuneus	147	-18	-72	34	5.12
T2DM<HCs	Left aHPC	Right precuneus	153	8	-58	38	-4.59
		Right posterior middle temporal gyrus	137	56	-46	0	-4.83
T2DACD>T2DM	Left pHPC	Left precuneus	111	-30	-68	38	4.24
T2DACD<T2DM	Right mHPC	Left precuneus	216	-16	-80	34	-5.16

All tests were corrected for multiple comparisons using cluster-level FWE corrected at p<0.05, voxel-level uncorrected at p<0.001, voxels size>50. HCs, healthy controls; T2DM, type 2 diabetes mellitus; T2DACD, type 2 diabetes mellitus-associated cognitive decline; aHPC, anterior hippocampus; mHPC, middle hippocampus; pHPC, posterior hippocampus.

### 3.3 Relationship between cognitive characteristics and RSFC

**Figure 5:**
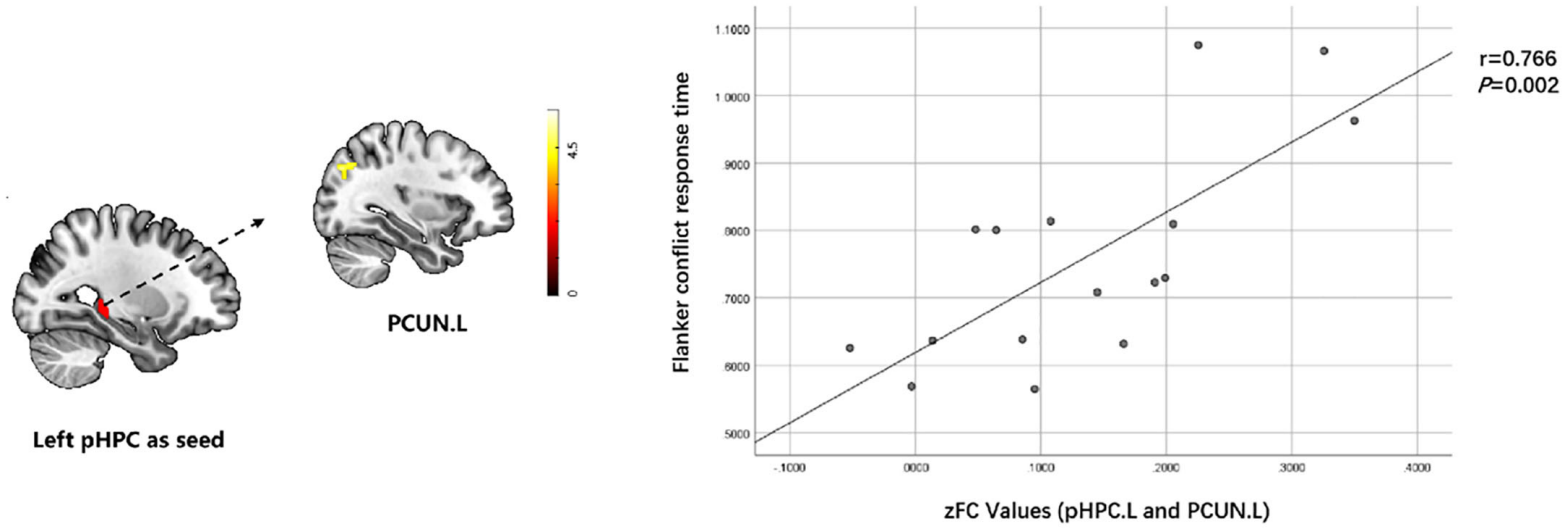
Relationship between cognitive characteristics and altered RSFC values in T2DACD. Flanker conflict condition response was positively correlated with RSFC of pHPC.L with PCUN.L in T2DACD. pHPC.L, left posterior hippocampus; PCUN.L, left precuneus; T2DACD, type 2 diabetes mellitus-associated cognitive decline; color bar, z value.

Flanker conflict condition response was positively correlated with RSFC of the left posterior hippocampus with left precuneus (r=0.766, *p*=0.002) in T2DACD (**Figure 5**). However, there was no significant relationship between the results of the other cognitive assessment and RSFC z-values of significantly altered brain clusters.

### 3.4 ROC curves results

**Figure 6:**
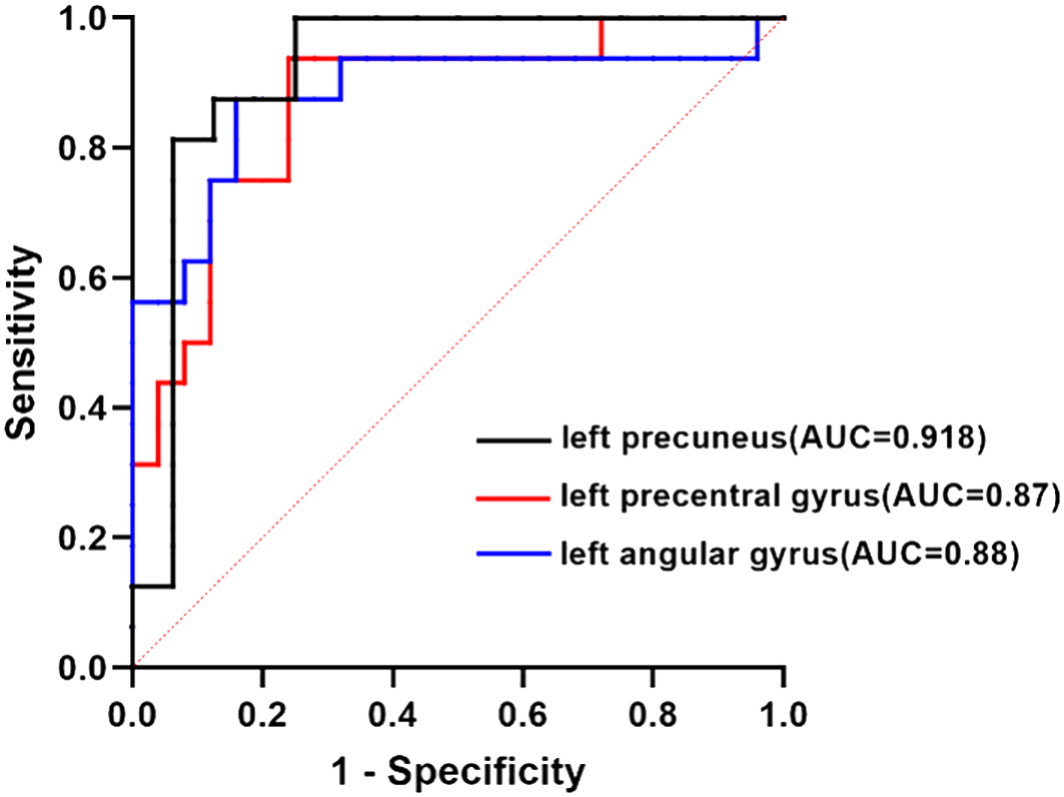
ROC curve analysis. ROC curve for altered RSFC patterns based on the left anterior hippocampus and left posterior hippocampus to distinguish T2DACD from HCs or T2DM. T2DACD, type 2 diabetes mellitus-associated cognitive decline; HCs, healthy controls; T2DM, type 2 diabetes mellitus.

Using the values of AUC and 95% CI, the RSFC z-values determined between the left posterior hippocampus and left precuneus showed the most accurate classification. Using the AUC, it could be found that the RSFC z-values between the left posterior hippocampus and left precuneus, left anterior hippocampus with left precentral gyrus or left angular gyrus all reached a significant level of *p* < 0.0001 (**Table 6**, **Figure 6**).

**Table 6:** The ROC analysis for altered brain regions that distinguish T2DACD from HCs or T2DM.

Comparison	Brain regions	SEN	SPE	AUC	95% CI	*p* value	YI
T2DACD vs HCs	**left anterior hippocampus**						
	zFC_left precentral gyrus	0.938	0.760	0.870	0.756-0.984	**<0.0001**	0.698
	zFC_left angular gyrus	0.875	0.840	0.880	0.755-1.000	**<0.0001**	0.715
T2DACD vs T2DM	**left posterior hippocampus**						
	zFC_left precuneus	0.8125	0.9375	0.918	0.807-1.000	**<0.0001**	0.75

SEN, sensitivity; SPE, specificity; AUC, area under the ROC curve; CI, confidence interval; YI, Youden’s index; T2DACD, type 2 diabetes mellitus-associated cognitive decline; T2DM, type 2 diabetes mellitus; HCs, healthy controls; zFC, functional connectivity z values. Statistically significant results (i.e., p<0.0001) appear in boldface type.

## 4 Discussion

The results of this study found that compared with HC, T2DACD and T2DM both showed an enhanced peripheral inflammatory response, manifested by an increase in the expression of IL-6 in the peripheral serum. T2DACD exhibits cognitive decline and abnormalities RSFC in the left anterior hippocampus, with different brain regions including the left precentral gyrus and left angular gyrus. T2DM did not show a significant cognitive decline, but abnormalities RSFC in the bilaterally anterior and middle hippocampus had occurred, with differential brain regions including the right medial frontal gyrus, right middle temporal gyrus posterior and bilateral precuneus. In addition, abnormalities RSFC in the left posterior hippocampus and right middle hippocampus occurred in T2DACD compared with T2DM, and differential brain regions were all in the left precuneus. The RSFC in the left posterior hippocampus with left precuneus of T2DACD was positively correlated with the completion time of the Flanker conflict test. In the ROC analysis, the RSFC of T2DACD was significant between the left posterior hippocampus and left precuneus, left anterior hippocampus with left precentral gyrus or left angular gyrus. Based on these findings, T2DACD may have underlying dysfunction in the hippocampal subregion with DMN and sensory-motor network (SMN), and T2DM may have dysfunction primarily in the hippocampal subregion with DMN. The left precuneus may be an important brain region in the pathological transition from T2DM to T2DACD, which may be closely related to decreased attentional control in T2DACD patients. The DMN and SMN are associated with cognition, memory, emotion and sensory control (42, 43). Therefore, prospective studies with large samples and high quality should be continued in the future to observe brain imaging, cognitive control, and memory-emotion.

The results of the clinical cognitive assessment showed that the MoCA scores of T2DACD were significantly lower than HCs and T2DM, suggesting that patients with T2DACD had experienced significant overall cognitive decline. MoCA is specifically developed for screening MCI (38), in terms of detection of cognitive impairment with good sensitivity and specificity (44). Specifically, MoCA can assess the visual space executive function, named ability, memory, attention, language features, abstraction and orientation ability (38). A previous systematic review and meta-analysis found the MoCA to be the most commonly used scale for examining the state of cognitive impairment in patients with T2DM, and the results of the study suggest that the global prevalence of T2DACD is high and should be of concern to primary care clinicians (45). In addition, T2DACD showed a significant increase in response time length for completing Flanker conflict, Stroop neutrality, Stroop conflict and 1-back. The Flanker task is to evaluate the standard tool for selective attention (46), selective attention both to focus on the information related to the target, including inhibition of conflict has nothing to do with the goal of information. Existing research has indicated that MCI has more difficulties in resolving inconsistent trials, and they show slower reaction times and lower accuracy than the healthy control group (47). The Stroop test is regarded as one of the gold standards in attention assessment, aimed at evaluating executive control and linguistic abilities (48). Previous studies have found, through the Stroop and Flanker tasks, that error rates are higher in patients with mild AD than normal aging population, and inhibitory control deficits occur more frequently in patients with mild cognitive impairment (49). The N-back task is one of the most frequently employed experimental paradigms for exploring the neural underpinnings of working memory and executive control. It serves as a critical method for assessing working memory deficits in patients with AD and MCI (50). Research indicates that the N-back task aids in the early diagnosis of MCI and offers an assessment of the neuropsychology in MCI (51, 52). The results of this study illustrate that attentional inhibition and working memory capacity are significantly reduced in T2DACD.

The detection of inflammatory factors in peripheral serum revealed elevated levels of IL-6 in both T2DACD and T2DM, with a tendency for T2DACD to be higher than T2DM. Under physiological conditions, IL-6 levels in the central nervous system are low, while their levels are elevated during brain injury, inflammation, and disease. IL-6 from peripheral blood can also flow into the brain parenchyma through the blood-brain barrier, leading to increased production of central inflammatory cytokines (53). However, there is evidence that IL-6 may also have anti-inflammatory and immunosuppressive properties that regulate neuronal survival and function at low levels (54). Several studies have found elevated IL-6 concentrations in brain tissue and peripheral blood in a variety of disorders, including AD, Lewy body dementia, and vascular dementia (55–57), suggesting that inflammatory mechanisms may be involved in the development of cognitive impairment. One study found an inverse relationship between blood IL-6 concentration and hippocampal gray matter volume (58). The results of this study indicate that both T2DACD and T2DM have peripheral inflammatory reactions, and T2DACD tends to be more severe than T2DM, which may also be one of the reasons affecting cognitive decline. In addition, IL-10 is an anti-inflammatory mediator that has been noted to be one of the major cytokines associated with the development of AD (59). TNF-α is considered an inflammatory substrate for cognitive impairment in the early clinical stages of dementia (60), and studies have found that TNF-α-targeted therapies may be a biologically feasible way to maintain cognitive ability (61). However, in the results of this study, no significant abnormalities of IL-10 and TNF-α expression in the peripheral serum of T2DACD and T2DM were detected, which may require further observation by increasing the sample size to validate the results. In addition, existing studies have found that central inflammatory processes lead to hippocampal inhibition and neuronal apoptosis (62). Therefore, it is still necessary to explore the specific relationship between T2DACD and inflammatory cytokines through basic research in the future.

RSFC analyses showed increased RSFC in the left anterior hippocampus with left precentral gyrus and decreased RSFC in the left anterior hippocampus with left angular gyrus in T2DACD compared with HCs. The anterior hippocampus has extensive connectivity related to emotional responses, spatial navigation, and imagination (22, 63). The study confirms that the anterior hippocampus is normally co-activated with brain regions associated with the somatomotor network in the resting state (64). The precentral gyrus is located in the frontal lobe and belongs to the primary motor cortex, which is mainly associated with motor learning, motor memory and motor control (65, 66). The primary motor cortex is not only a structure that controls movement, but also a dynamic substrate that may also contribute to motor learning and cognitive processes (67). The angular gyrus is the visual language center in the brain. Clinical impairment of the angular gyrus results in dyslexia, where there is no visual impairment but the person is unable to read the original literate person (68). The research findings may speculate that patients with T2DACD may show abnormalities in the ability of recognize, interpret or remember the words they see compared to normal individuals. Previous studies have also found that decreased RSFC of the hippocampal cognitive subregions with angular gyrus is associated with decreased cognitive ability (69). In contrast, perceptual-motor brain regions are compensatorily enhanced to compensate vicariously for deficits in perceptual and cognitive processing abilities. Compared with T2DM, RSFC was increased in the left posterior hippocampus with left precuneus and decreased in the right middle hippocampus with left precuneus in T2DACD. Studies have shown that the middle/posterior hippocampus primarily performs cognitive functions (17). In the resting state, the middle/posterior hippocampus is usually co-activated with brain regions associated with the DMN (64). The precuneus, as a component of the DMN, is associated with a variety of cognitive functions, including processes such as situational memory, visuospatial, and information processing (64). One study found that the precuneus often referred to as a remote association node of the hippocampal intrinsic connectivity network (70, 71), which is a key abnormal brain region in the episode memory deficits observed early in AD (72, 73). Recent studies have found that precuneus magnetic stimulation may slow down cognitive and functional decline in AD (74). The results of this study may speculate that the detection of abnormal precuneus activity may help in the early clinical application of imaging to identify the possibility of cognitive decline in patients with T2DM. In this study, the Flanker conflict condition response patients was positively correlated with RSFC of the left posterior hippocampus with left precuneus in T2DACD. The longer Flanker conflict response time, the worse cognitive control ability of T2DCD patients. These results indicated that the worse cognitive control ability of T2DACD patients, the higher RSFC connectivity strength of the left posterior hippocampus with left precuneus. A recent study found that 2 weeks of rTMS stimulation of the precuneus improved connectivity in the posterior hippocampus, potentially resulting in an improvement of cognitive memory in patients with cognitive decline (75). To some extent, this finding is consistent with ours. The precuneus-posterior hippocampus may be key to improving cognitive memory and preventing the progression of cognitive disorders.

T2DM compared with HCs, RSFC was increased in the left middle hippocampus with right medial frontal gyrus and right anterior and middle hippocampus with left precuneus, decreased RSFC in the left anterior hippocampus with right precuneus and right posterior middle temporal gyrus. In this study the abnormal brain regions of T2DM, precuneus, middle temporal gyrus, and medial frontal gyrus were all important constituent brain regions of DMN. Some studies have confirmed that DMN is closely related to a variety of brain functions such as self-awareness, self-cognition, social cognition and spatial perception. Abnormalities in DMN function can mostly be seen in normal brain aging and cognitive disorders related disorders, and it is considered to be the most promising factor for neuroimaging (76–78). Recent studies have found that the change of dynamic functional connectivity pattern in the hippocampal subregions and default mode network, sensorimotor network or visual function network may be related to the decline of cognitive function (79). The present study demonstrates that the hippocampal subregions of T2DM had RSFC with the DMN and that the differences in the bilateral hippocampus manifest differently. Although patients with T2DM did not show significant differential changes in cognitive-behavioral functioning, the RSFC of brain regions has been abnormal, which further indicates that T2DM is an important risk factor for MCI and AD.

Previous studies on diseases related to cognitive decline have examined the hippocampus as a whole, ignoring its structural and functional complexity and limiting the diversity of findings. This study preliminarily explored a new pattern of RSFC in the hippocampal subregion of T2DACD, which enhances the accuracy of the findings and provides a new way of thinking to study the pathogenetic features of T2DACD. However, there are still some limitations of this study that need to be further addressed. First, the sample size of this study was small, and there is a need to increase the sample size to verify the generalizability of the current results. Second, the relatively small amount of data used to assess the clinical characteristics of the patients should be increased by looking at multiple cognitive dimensions, such as memory, language, visuospatial, executive, computational and comprehension judgments, to make the findings more comprehensive. Third, the present study only explored the abnormal alterations of the functional networks in the hippocampal subregion of T2DACD, and there is a need to further explore the characteristics of more cognitively relevant brain network changes (DMN, cognitive control network, dorsal attention network). Fourth, this study only initially explored the brain abnormalities of T2DACD, and further basic research is necessary to investigate the biological basis of the alterations in brain regions.

## 5 Conclusion

In this study, we examined RSFC patterns in hippocampal subregions of T2DACD and T2DM patients for the first time. The findings showed that both T2DACD and T2DM exhibit enhanced peripheral pro-inflammatory response. Patients with T2DACD exhibit cognitive decline similar to MCI and AD, and there may be underlying dysfunction of DMN and SMN in the brain. T2DM did not show cognitive decline, but dysfunction of DMN has occurred in the brain. The left precuneus may be an important brain region in the pathologic transition from T2DM to T2DACD. The results of this study may provide a new basis for the clinical diagnosis of T2DACD. Future prospective studies with large samples and high quality should be continued to observe brain imaging, cognitive control, and memory-emotion.

## Data Availability

The raw data supporting the conclusions of this article will be made available by the authors, without undue reservation.
